# Interpreting Echocardiographic Right Ventricular-Pulmonary Arterial Coupling Indices After Transcatheter Tricuspid Intervention

**DOI:** 10.1016/j.shj.2026.100842

**Published:** 2026-06-23

**Authors:** Gianluca Pagnoni, Francesco Marangi, Aurora Vicenzi, Francesca Coppi

**Affiliations:** Cardiology Unit of Emergency Department, Guglielmo da Saliceto Hospital, Piacenza, Italy; Department of Medical and Surgical Sciences for Children and Adults, University of Modena and Reggio Emilia, Modena, Italy; Department of National Institute for Cardiovascular Research (INRC), Bologna, Italy; Department of Medical and Surgical Sciences for Children and Adults, University of Modena and Reggio Emilia, Modena, Italy

Dear Editor,

We read with great interest the article entitled “Comparison of Echocardiographic Right Ventricular Pulmonary-Arterial Coupling Indices in Patients Undergoing Transcatheter Tricuspid Valve Repair”,^1^ which investigates the prognostic and functional relevance of different echocardiographic indices of right ventricular–pulmonary arterial (RV–PA) coupling in patients undergoing transcatheter tricuspid valve repair (TTVR). The study addresses a clinically relevant and timely topic given the growing recognition of right-sided heart failure and the expanding role of transcatheter therapies for tricuspid regurgitation.

The authors should be commended for focusing on RV–PA coupling, which has increasingly been recognized as a central determinant of symptoms, functional capacity, and prognosis in patients with right ventricular dysfunction and pulmonary vascular disease. The comparative assessment of multiple echocardiographic indices represents the strength of the study and highlights the complexity of right ventricular–arterial interactions in this setting.

Nevertheless, some considerations deserve further discussion. RV–PA coupling parameters derived from resting echocardiography are intrinsically load-dependent. Following TTVR, the abrupt reduction in tricuspid regurgitation induces significant changes in right ventricular preload and afterload, potentially influencing conventional functional indices independently of intrinsic myocardial adaptation. This phenomenon has been extensively described in both experimental and clinical studies and represents a major limitation when interpreting single time point measurements after valvular intervention.^2^

Moreover, RV–PA coupling is a dynamic property rather than a static condition. Several studies have demonstrated that right ventricular dysfunction and uncoupling may become evident only during physical stress, even when resting parameters appear preserved. Exercise or stress-based assessment has therefore been proposed as a valuable tool to unmask latent RV–PA uncoupling and improve risk stratification, particularly in patients with pulmonary hypertension or advanced right-sided heart failure.^3^

In this regard, integrating echocardiographic coupling indices with functional evaluation may enhance their clinical relevance. Functional capacity and exercise tolerance are strong prognostic markers in cardiovascular disease, and adherence to structured exercise and rehabilitation programs has been shown to significantly influence clinical outcomes and recovery trajectories.^4^ The combination of imaging-derived RV–PA coupling parameters with functional endpoints may therefore provide a more comprehensive assessment of patient status after TTVR.

Furthermore, indices such as the tricuspid annular plane systolic excursion/pulmonary artery systolic pressure (TAPSE/PASP ratio have demonstrated prognostic value across different disease states characterized by pulmonary vascular involvement, including systemic sclerosis–associated pulmonary hypertension and other forms of pulmonary hypertension.^5^ These data support the broader applicability of RV–PA coupling metrics while also underscoring the need for cautious interpretation in the presence of significant changes in loading conditions.^6^

In conclusion, this study represents an important contribution to the understanding of RV–PA coupling in patients undergoing TTVR. Future research integrating resting and stress-based assessments, functional capacity measures, and longitudinal evaluation may further refine the clinical utility of echocardiographic coupling indices and support their implementation in routine practice.

## Declaration of Generative AI and AI-Assisted Technologies in the Writing Process

An artificial intelligence–based language model was used to assist with language editing and structural refinement of this manuscript.

## Funding

The authors have no funding to report.

## Disclosure Statement

The authors report no conflict of interest.
